# Seed Oil from Mediterranean Aromatic and Medicinal Plants of the Lamiaceae Family as a Source of Bioactive Components with Nutritional

**DOI:** 10.3390/antiox9060510

**Published:** 2020-06-10

**Authors:** María Quílez, Federico Ferreres, Santiago López-Miranda, Eva Salazar, María J. Jordán

**Affiliations:** 1 Research Group on Rainfed Crops for the Rural Development, Murcia Institute of Agri-Food Research and Development (IMIDA), c/Mayor s/n, 30150 La Alberca Murcia, Spain; maria.quilez@carm.es; 2 Department of Food Technology and Nutrition, Molecular Recognition and Encapsulation (REM) Group, Universidad Católica de Murcia (UCAM), Avenida de los Jerónimos 135, Guadalupe, 30107 Murcia, Spain; fferreres@ucam.edu (F.F.); slmiranda@ucam.edu (S.L.-M.); 3 Department of Food Technology and Nutrition, Industrial Processing Technology and Cooking Food Group, Universidad Católica de Murcia (UCAM), Avenida de los Jerónimos 135, Guadalupe, 30107 Murcia, Spain; esalazar@ucam.edu

**Keywords:** Lamiaceae seed oil, fatty acid composition, tocopherol content, phenolic compounds, antioxidant capacity

## Abstract

The potential use as food ingredients of 12 commercial seed species belonging to the Lamiaceae family constitutes the main goal of this research. For that, the oil yield, the lipid profile, tocopherol content, phenolic profile and antioxidant capacities were determined. Seeds from *Satureja hortensis, S*. *montana*, *Lavandula angustifolia, L. latifolia* and *Origanum vulgare* can be considered as important sources of ω-3 polyunsaturated fatty acids (50.5; 52.4; 52.1; 48.5 and 45.5 g/100 g, respectively), likewise for seed oils from *Salvia lavandulifolia* and *Rosmarinus officinalis* regarding ω-6 polyunsaturated fatty acids (52.2 and 50.0 g/100 g, respectively). The total tocopherol concentration varied between 42.8 and 113.8 mg/100 g of oil. The highest antioxidant capacities corresponded to *Thymbra capitata* and *Origanum vulgare* seed oils, in which carvacrol was the major phenolic monoterpene quantified. The presence of cinnamoyl-apigenin derivatives and hydroxycoumarin-apigenin-derivatives in spike lavender is described for the first time. Most of these seeds can be considered as an important source of oil rich in bioactive components of interest for human nutrition.

## 1. Introduction

Nowadays, there is worldwide recognition to the importance of aromatic and medicinal plants not only on the bases of their curative and preventive properties but also for their uses in food, feed, cosmetics and pesticide industries, which is reflected by a growing demand for aromatic and medicinal products in the markets [1].

Given this market situation of growth and development, there is a need to continue researching these plants, to introduce new products, such as their seeds, which would help to increase the sustainability of their production. In this sense, as it occurs with the oleaginous plants, seeds of aromatic and medicinal plants could also be considered as a new source of oils rich in bioactive components.

Among the aromatic and medicinal plants, those belonging to the Lamiaceae family stand out, since their beneficial properties have been known since ancient times, and plants of the genera *Rosmarinus*, *Thymus*, *Salvia*, *Lavandula*, *Ocimum*, *Thymbra*, *Satureja*, *Origanum* and *Perilla*, among others, are currently exploited due to their medicinal, culinary and industrial characteristics. Despite the interest in the potential uses of these plants and their seeds in the food industry, little previous work has been done to characterize them as a source of bioactive compounds. Only *Salvia hispanica* (chia) has been the subject of detailed studies. Zettel and Hitzmann (2018) [2] published a recent review of the applications of chia in food products, detailing the uses in baked foods, dairy products, meat and fish products, gluten-free products and other functional foods. However, references related to the industrial utilization of other Lamiaceae seeds are scarce.

Among the species previously studied, perilla (*Perilla frutescens*) is of note since, according to Asif (2011) [3], its seed oil yield ranged between 35% and 45%, with a lipid profile rich in linolenic acid (ALA) (54–64%), linoleic acid (11–16%) and oleic acid (14–23%). In oregano seeds (*Origanum onites* L. and *O. vulgare*, with oil yields of 14.1–20.0% and 18.5%, respectively), 20 fatty acids were identified and quantified in their oils, and over 80% of this oil was represented by the polyunsaturated fatty acids (PUFA) fraction [4]. Shrub species, such as *Salvia* spp. [5] and *Satureja* spp. [6], have also exhibited lipid profiles rich in the PUFA fraction. For *Rosmarinus officinalis*, only one reference has been found pertaining to its foliar lipids: 50–60% of these were comprised by PUFA (of which α-linolenic acid and linoleic acid represented 32–42% and 21–28%, respectively), whereas palmitic acid represented 13–17% of the saturated fatty acids fraction [7].

The nutritional value of vegetable oils and their oxidative stability are determined by the presence of antioxidants, such as vitamin E, phytosterols, carotenoids and phenolics [8]. Vitamin E consists of four tocopherols, four tocotrienols and plastochromanol-8, collectively known as tocochromanols [9]. In order to prevent lipid oxidation, plants mainly accumulate tocochromanols in oily seeds and fruits or young tissues, which are undergoing intensive cell division. Thus, seeds accumulate tocochromanols, with γ-tocopherol as the main homologue [10,11]. Bioactive compounds including quercetin, kaempferol, catechin and caffeic, vanillic and chlorogenic acids have also been identified in chia seed oils [12].

The increasing interest in seed oil species is related to the exploitation of chia seeds, especially in the food industry [2]. This species has undergone, in recent years, a revaluation due to the interest in its nutritional properties mainly its seed oil, with high levels of PUFA (linoleic and linolenic) and a low content of saturated fatty acids [13]. As indicated by Ixtaina et al. (2011) [14], this seed is considered as one of the major sources of ALA, whose yield in chia seeds is estimated to be between 25% and 38% [15]. The European Food Safety Agency (EFSA), in 2009, approved the commercialization of this Lamiaceae seed as a food ingredient. Subsequently, in 2014, the marketing of chia oil was authorized [16]. Other example includes coriander seed oil (*Coriandrum sativum*), with a richness in monounsaturated fatty acids (MUFA) of 68–90% that is attributable mainly to its content of petroselinic acid (60–75%) (18:1 *n*-6) [17].

Taking into account the described background, the characterization of seeds of aromatic plants is necessary to establish new sources of oils rich in PUFA, vitamin E and phenolic compounds with a great interest for the human diet. Therefore, the main aim of this study was to perform a thorough characterization of the seed oils of the Lamiaceae species cultivated most in the western Mediterranean area, based on the hypothesis that some of them could be new and interesting sources of natural antioxidants for functional foods.

## 2. Materials and Methods

### 2.1. Seeds

Commercial seeds of species belonging to the Lamiaceae family including *Satureja hortensis* L. (summer savory), *Satureja montana* L. (winter savory), *Ocimum basilicum* L. (basil), *Origanum vulgare* L. (oregano), *Rosmarinus officinalis* L. (rosemary), *Salvia lavandulifolia* L. (Spanish sage), *Lavandula latifolia* Medicus. (spike lavender), *Lavandula angustifolia* subsp. *officinalis* (lavender), *Thymbra capitata* L. Cav. (Spanish oregano), *Thymus hyemalis* L. (winter thyme), *Thymus zygis* subsp. *gracilis* (red thyme) and *Thymus vulgaris* L. (common thyme) were purchased from Semillas Silvestres S.L. (Córdoba, Spain), and *Salvia hispanica* (chia) was acquired from a local supermarket (Murcia, Spain).

Most of these species grow spontaneously in the Spanish Mediterranean area, and currently they are exploited at the commercial level as condiments (dry leaves) and/or for the extraction of their essential oils. Seeds of *S. hispanica*, native to tropical regions, were included as a standard of reference in this study due to the increasing interest in this species, at both technological and nutritional levels, regarding its lipid and nutritional properties.

### 2.2. Extraction of Seed Oil

The lipid content was determined after Soxhlet extraction using a BÜCHI Extraction System B-811 (Buchi, Flawil, Switzerland). Powdered seeds (3 g) were extracted using a mixture of n-hexane and ethyl acetate (6:4, V/V) and, after 4 h of distillation, samples were dried at 40 °C, under vacuum in an evaporator system (Syncore Polyvap R-96) (Buchi, Flawil, Switzerland). The yield percentages are expressed as gram of oil per 100 g of dry seed.

In order to avoid damage caused by the high temperatures to some active compounds, an alternative seed-oil extraction procedure was used for the lipid profile, vitamin E, phenol content and antioxidant activity determinations. Seed oils were isolated using a liquid extraction technique. Powdered seeds (3 g) were mixed with 40 mL of n-hexane-ethyl acetate (6:4, V/V). The oil fraction was extracted by stirring for 4 h with a magnetic stirrer (OVAN MultiMix D, Barcelona, Spain) at 15 °C, in dark and in an inert atmosphere. The water content was removed from the mixture by the addition of anhydrous sodium sulphate and centrifugation in an Eppendorf 5810R Centrifuge (Eppendorf AG, Germany) at 2061 g for 10 min. The supernatant was filtered through a 0.45-µm filter (Millipore SAS, Molsheim, France) and was dried at 35 °C under vacuum, in an evaporator system (Syncore Polyvap R-96) (Buchi, Flawil, Switzerland). The oils were kept in vials at −80 °C until their corresponding analysis was done.

### 2.3. Gas Chromatography–Mass Spectrometry Analysis

Fatty acids were converted into fatty acid methyl esters (FAMES) by using the general method of trans-methylation/methylation. This esterification was performed under sequential conditions, catalysed by alkali and by acid, respectively, as described in the UNE-EN ISO 12966-2: 2017 standard [18]. The FAMES were analysed using an Agilent Technologies (Santa Clara, CA, USA) 6890 N gas chromatograph (GC) equipped with a 30 m × 0.25 mm i.d. DB-23 (50% cyanopropyl-methylpolysiloxane) column with a film thickness of 0.25 µm. The column was supplied by Agilent Technologies (Santa Clara, CA, USA). Helium was used as the carrier gas (constant pressure, nonadecanoic acid methyl ester eluting at 13.33 min), and the split ratio was set to 100:1, with 1 µL of injected sample. The GC was linked to an Agilent model 5972 inert mass spectrometry detector. The initial oven temperature was set at 50 °C and then, it was increased at the rate of 25 °C/min to 175 °C, followed by an increase of 4 °C/min until 211 °C, then up to 216 °C at 1 °C/min and finally, it was raised to 230 °C at a rate of 5 °C/min. The injection port and the transfer line to the mass selective detector were kept at 250 °C and 280 °C, respectively. The mass spectrometer was operated in electron impact ionization mode with an ionizing energy of 70 eV, scanning from *m*/*z* 45–425 at 3.21 scan/s. The quadrupole temperature was 150 °C and the electron multiplier voltage was maintained at 1300 V. The identification of the individual compounds was made by considering the retention times relative to those of pure reference standards and by comparison of mass spectra using the NBS75K (US National Bureau of Standards, 2002) library and the spectra obtained from the standards.

For the absolute quantification, linear regression models were applied using calibration standard solutions, with nonadecanoic acid methyl ester as an internal standard. Target ions were used in the absolute quantification of each component by mass spectrometry. High-purity standards were purchased from Sigma-Aldrich (Madrid, Spain).

### 2.4. Tocopherol Content

The analysis of the tocopherols profile in the seed oils was carried out using an HPLC 1200 (Agilent, Waldbronn, Germany) system equipped with a G1311A binary pump and two detectors, a G1315A photodiode array UV/Vis (wavelengths of detection were set at 292, 296, 298 and 262 nm) and a G1321A FLD programmed for excitation at 290 nm and emission at 330 nm [19]. Samples were diluted in acetone (25–50 mg/mL) and 15 µL were injected onto a reverse ZORBAX SB-C18 column (4.6 × 250 mm, 5-µm pore size, Agilent Technologies, Santa Clara, CA, USA) using a guard column (ZORBAX SB-C18 4.6 × 125 mm, 5-µm pore size, Agilent Technologies, Santa Clara, CA, USA), with a flow rate of 1 mL/min at 25 °C. The mobile phase, pumped at 1 mL/min, was methanol (A) and tert-butyl methyl ether (B). The gradient applied was as follows: 0 min, 99% A; 3 min, 98% A; 6 min, 97% A; 9 min, 96% A; 12 min, 95% A; 25 min, 89% A; 28 min, 75% A; 30 min, 99% A and 35 min, 99% A. Individual peaks were identified and quantified using calibrations curves of the corresponding standard compounds. High-purity standards were purchased from Sigma-Aldrich (Madrid, Spain).

### 2.5. Phenolic Profile

#### 2.5.1. HPLC-DAD-ESI (Ion Trap)/MS Qualitative Analysis

Chromatographic analyses were carried out according to the method described by Ferreres et al. (2014) [20], with some modifications. In this case, the chromatographic column used was a Zorbax SB-C18 (5 µm, 4.6 × 250 mm; Agilent, Santa Clara, CA, USA) with a Security Guard ULTRA Cartridges (ZORBAX SB-C18, 4.6 × 125 mm, 5-µm pore size; Agilent Technologies, Santa Clara, CA, USA). The mobile phase consisted of acidified water (formic acid, 0.05%) (A) and acetonitrile (B); the gradient of the eluents started with 5% B, reaching 15% B at 10 min, 25% B at 30 min, 30% B at 35 min, 55% B at 50 min and 90% B at 60 min, with a flow rate of 1 mL/min. The UV detection was set at 280 and 340 nm. The injection volume was 20 µL, and the full scan mass covered the range from *m*/*z* 100 to 1000.

#### 2.5.2. UPLC-ESI-QTOF/MS Qualitative Analyses

For determination of the qualitative polyphenolic profiles, the seed-oil extracts were also analysed in an Agilent 1290 Infinity LC System coupled to the 6550 Accurate-Mass QTOF (Agilent Technologies, Waldbronn, Germany) with an electrospray interface (Jet Stream Technology), following the methodology of Garcia et al. [21] (2016). Samples (2 µL) were injected onto a reverse-phase Kinetex column (1.7 µm, C18, 100 A, 50 × 2.1 mm; Phenomenex, Macclesfield, UK) with a SecurityGuard ULTRA cartridges of the same material, operating at 30 °C and a flow rate of 0.5 mL/min. The mobile phases used were acidified water (0.1% formic acid) (A) and acidified acetonitrile (0.1% formic acid) (B). Compounds were separated using the following gradient conditions: 30% B initially, to obtain 60% B at 15 min and 90% B at 17 min. The optimal conditions for the electrospray interface were gas temperature 280 °C, drying gas flow 11 L/min, nebulizer pressure 45 psi, sheath gas temperature 400 °C, sheath gas flow 12 L/min and collision energy 40 eV. The MS system was operated in negative ion mode. The mass range was set at *m*/*z* 50–1000 in full scan resolution mode.

#### 2.5.3. HPLC-DAD Quantitative Analysis

For the purpose of quantifying the phenolic profile, the method published by Jordan et al. (2013) [22] was applied, using the chromatograph and the analytical column previously described for the tocopherol content analysis. The seed oils were diluted in acetone at a concentration of 200 mg/mL. Before injection, samples were filtered through a 0.45-µm filter (Millipore SAS, Molsheim, France). Samples (20 µL) were injected and the elution was performed at 25 °C. The mobile phase eluents were acetonitrile (A) and water acidified with 0.05% formic acid (B). The flow rate was 1.0 mL/min and the detection wavelengths were set at 280 and 330 nm. The concentrations of the polyphenolic components, determined using standard dilution techniques and their corresponding linear regression models, were expressed in milligram per gram of oil. High-purity standards were purchased from Sigma-Aldrich (Madrid, Spain).

### 2.6. Antioxidant Activity

#### 2.6.1. DPPH^•^ Radical-Scavenging Activity

The scavenging activities of the seed oils were determined according to the method described by Krzyczkowska and Kozlowska (2017) [23], with some modifications. Samples were diluted in ethyl acetate at a concentration of 33 mg/mL. Next, 250 µL of the oil solutions were added to 250 µL of methanol and 1 mL of a methanolic solution of DPPH^•^ (0.1 mM). The mixtures were vortexed and, after incubation for 30 min at room temperature in the dark, the absorbance was measured at 517 nm in a spectrophotometer (Shimadzu UV-VIS2040 PC, Tokyo, Japan). The calibration curve of Trolox was linear in the range of 25–230 µM. The results were expressed as micromole of Trolox equivalents/gram of oil sample.

#### 2.6.2. ABTS^•+^ Radical Cation Decolouration Assay

The ABTS radical-scavenging activity was determined according to the method of Re et al. (1999) [24]. The ABTS (2,2-azinobis-(3-ethylbenzothiazoline-6-sulphonic acid) radical solution was prepared by mixing ABTS solution (14 mM) and an equal volume of 4.9 mM potassium persulphate solution (final concentration was 7 mM ABTS in 2.45 mM potassium persulphate). The mixture was left in the dark at room temperature for 12 to 16 h before use. The ABTS solution was diluted with 95% ethanol to an absorbance of 0.70 ± 0.02 at 734 nm and equilibrated at 30 °C. Then, 15 μL of diluted seed oil (200 mg/mL in acetone) were added to 1.5 mL of diluted ABTS radical solution. The mixture was vortexed and left in the dark for 3 min prior to measurement at 734 nm against a blank. The regression curve was linear in the range of 300–2500 µM Trolox. The results are expressed as micromole of Trolox equivalents per gram of seed oil.

#### 2.6.3. Oxygen Radical Absorbing Capacity (ORAC)

The automated oxygen radical absorbing capacity (ORAC) assay was carried out with a Synergy HT Multi-Detection Microplate Reader, from BioTek Instruments, Inc. (Winooski, VT, USA), using 96-well polystyrene microplates, as described by Davalos et al. (2004) [25]. For the reaction, 20 µL of diluted seed oil (5 mg/mL in acetone) was added to 50 µL of 75 mM sodium phosphate buffer (pH 7.4) and 100 µL of fluorescein (6 nM). The mixture was preincubated for 30 min at 37 °C and, after this, 30 µL of AAPH (150 mM) was added to each well of the microplate. Fluorescence readings (excitation 485/20 nm and emission 528/20 nm) were recorded every 74 s for 120 min, when the fluorescence loss reached 95%. For the quantification, a standard curve was obtained by plotting eight concentrations of Trolox standards (from 3.12 to 31.25 μM) as antioxidants. The final ORAC values were expressed as micromole Trolox equivalents per gram of seed oil.

### 2.7. Statistical Analysis

Statistical analyses were performed based on the three samples and three analytical replicates of each sample. A one-way ANOVA was carried out to assess the significant differences (a model was accepted as significant for a *p*-value < 0.05), using the IBM SPSS Statistics Program (v. 25). Next, Fisher’s LSD pairwise comparison was performed on the data.

## 3. Results and Discussion

Due to their adequate PUFA (ω-6/ω-3) ratio, chia seeds are one of the seeds most in demand, commercially, either as a food or as a functional ingredient. Taking this into account, the results of chia seeds have been considered as a reference for the analysis and discussion of the rest of the seed oils under study here.

### 3.1. Oil Yield and Lipid Profile

**Figure 1:**
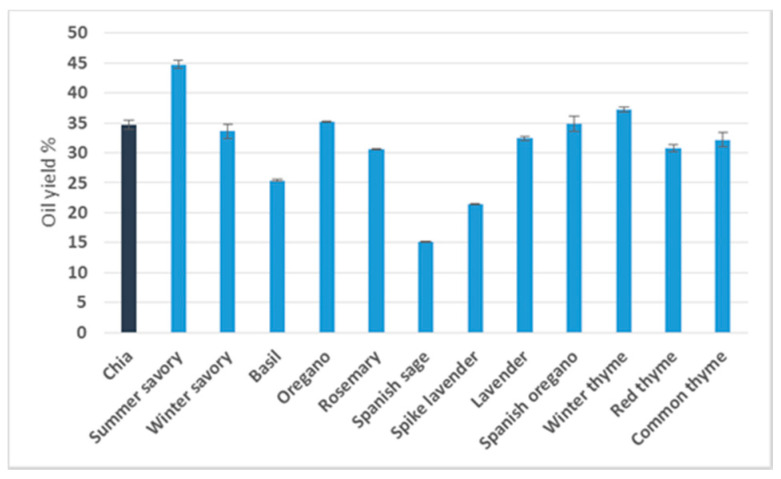
Oil yields (g oil/100 g dry seeds) from the Lamiaceae seeds under study.

Regarding the lipid content, as can be seen in Figure 1, the yields ranged from 15.1% to 44.8%. Among the seeds analysed, the most productive, regarding this parameter, were those of summer savory (44.8%) and winter thyme (37.2%), with values that exceeded, to a statistically significant extent, those obtained for chia seeds (34.7%). Such high yields have also been reported in *Perilla frutescens,* a Lamiaceae species with a lipid content that varied between 35% and 45% [3]. In our study, a fat content close to that of chia was found in seeds of oregano, Spanish oregano and winter savory. The species that yielded the lowest level of oils were spike lavender (21.4 %) and Spanish sage (15.1%). This last value was expectable since similar results (15–23%) were obtained by Ben Farhat et al. (2015) [5] for different subspecies of this Lamiaceae species. It is also interesting that the fat yield obtained from oregano (35.2%) was around double that reported by Azcan et al. (2004) [4] for *Origanum onites* and *Origanum vulgare* (14.1% and 18.5%, respectively). Besides, for lavender, the yield obtained (32.4%) was higher than those reported by Matthäus et al. (2015) [19], with values ranging from 18.1% to 28.7%.

This information points out the richness of some of these unexploited seeds as a source of oil, and their lipid profiles have also been considered.

Given the fact that the seed ethereal extracts are mainly composed of fatty acid triesters linked to glycerol molecules, together with fat-soluble vitamins, carotenes, waxes, resins and sterols in a greater or lesser proportion, in terms of productivity and functional properties, it is necessary to calculate the absolute concentration of every fatty acid in this lipid fraction. Based on this, results concerning the absolute quantification of every fatty acid present in 100 g of oil are shown in Table 1.

Regarding the saturated fatty acids (SFA) profile, as a general pattern, most of the oils analysed exhibited values for this lipid fraction lower than that of chia (8.0 g/100 g), with the exception of rosemary, which showed levels similar to that of the chia oil used as a reference. Although, it is interesting that, in the cases of basil, Spanish sage, rosemary and Spanish oregano, the concentrations of palmitic acid and stearic acid did not show statistically significant differences with respect to those quantified in chia oil (Table 1). The seed oils that showed the lowest concentrations of SFA corresponded to the savory and thyme species under study, their concentrations being almost 50% lower than those described for the other oils.

According to the specifications published by the United States Department of Agriculture [26], in chia seeds, the concentrations of palmitic and stearic acids are directly proportional and are maintained at a ratio close to 2:1. This same pattern, with the exception of Spanish sage, seemed to be generalized among all the seeds studied. These results, together with the low levels of C18:0 stearic acid and C20:0 arachidic acid, indicate that these seed oils have a low content of SFA, with a positive connotation in terms of human health.

Considering the MUFA fraction, a modest contribution to the lipid profile was quantified in all the seed oils under study (Table 1). Oleic acid was the major MUFA quantified, in contrast to vaccenic acid that was detected at low concentrations; in addition, it is described for the first time as part of the fatty acid lipid profile of winter, red and common thyme species. The presence of vaccenic acid in plants of the Lamiaceae (oregano, sage, spike lavender, lavender and summer savory) was previously reported by Azcan et al. (2004) [4] and Matthäus et al. (2015) [19]. Among the oils analysed, rosemary and Spanish sage had the highest oleic acid concentrations (16.2 and 16.3 g/100 g, respectively). For sage, this value was expected since Matthäus et al. (2015) [19] reported similar levels for *S. officinalis*, but it is higher than that described by Ben Farhat et al. (2015) [5] for the *Salvia* species *vellerea*, *officinalis* and *aegyptiaca*. The interest in the ω-3 and ω-6 PUFA, as a source of essential bioactive components, derives from the influence of an adequate intake of these ω-3 acids (linolenic and stearidonic) and ω-6 acids (linoleic and ϒ-linolenic) on the correct functioning of the metabolic pathways. These PUFA act as precursors of the long-chain PUFA, including eicopentanoic acid (EPA), docohexanoic acid (DHA) and arachidonic acid (AA), which in turn are precursors of eicosanoids and docosanoids with elevated biological activity [27].

Related to the ω-3 PUFA, encouraging results were obtained (Table 1) for summer savory (48.3 g/100 g), winter savory (50.2 g/100 g), lavender (49.8 g/100 g), spike lavender (46.5 g/100 g) and oregano (43.2 g/100 g) seed oils, since they exhibited absolute ALA concentrations even higher than those quantified in chia (42.5 g/100 g), used as a nutritional reference in this study. In line with this, other ω-3 PUFA, tentatively and exclusively identified in the *Thymus* species under study here, correspond to heptadecatrienoic acid C17:3 n3 and 2-hydroxy octadecatrienoic acid C18:3 n3*^3^. These fatty acids, reported for the first time in these thyme species, were present at low concentrations, 1.0, 0.6 and 1.8 g/100 g for heptadecatrienoic acid and 3.9, 1.9 and 4.7 g/100 g for 2-hydroxy octadecatrienoic acid in winter, red and common thyme, respectively.

The contents of linoleic acid (the main component of the ω-6 PUFA family), shown in Table 1, differentiate two tendencies among the fatty acid seed profiles under study. The first comprises seeds with a high linoleic acid content, including sage and rosemary with 52.2 and 50 g/100 g, respectively, and the other seeds with a low content, such as the lavender and thyme species with levels close to 10 g/100 g. The high linoleic acid concentrations found in sage and rosemary are comparable to those quantified previously by Patterson et al. (2012) [28] in safflower, sunflower, soya and corn oils of 74, 60.2, 53.4 and 50 g/100 g of the edible portion, respectively. The seed oils of basil and Spanish oregano had concentrations of this essential fatty acid, linoleic acid, close to that of chia, the nutritional seed reference (17.3 g/100 g).

From these results, it can be concluded that seed oils from summer and winter savory, lavender, spike lavender and oregano are important sources of ω-3 PUFA, while seed oils from sage and rosemary are important sources of ω-6 PUFA. The absolute concentrations reached, in both cases, exceeded those detected in commercial chia seeds.

### 3.2. Tocopherol Content

Continuing with the parameters that define the nutritional value of a vegetable oil, it is also important to determine the vitamin E content. In the present study, the stationary phase used in the analysis of these tocochromanols forms did not separate the β and γ isomers. But, attending to Gruszka and Kruk (2007) [11], the scarce presence of β-tocopherol and the absence in nature of β-tocotrienol forms justify the quantification of just the δ and γ isomers as an indicator of the vitamin E content. Taking this into consideration, the tocochromanol contents in these Lamiaceae seed oils are shown in Table 2.

The total tocopherol content ranged from 42.8 mg/100 g of seed oil in rosemary to 113.8 mg/100 g of seed oil in basil. In all the seed oils under study, γ- and δ tocopherols were identified, the γ isomer being the one quantified at higher concentrations.

α-Tocopherol was not detected in chia oil, contrary to oregano, winter savory, rosemary, lavender and common thyme, in which its concentration ranged from 1.9 to 4.6 mg/100 g of oil. The absence of α-tocopherol in chia was also reported by Trela and Szymańska (2019) [8], who also found that the γ isomer was the homologue present at the highest concentration. However, controversy about the presence of α-tocopherol can be found in the scientific literature. Thus, Özcan et al. (2019) [12] reported a content of 51.17 mg/100 g of α-tocopherol in chia oil, which exceeded in abundance by β-tocopherol (67.8 mg/100 g). In all these cases, chia oil was obtained using a cold-press system and the differences could be attributed to the different geographical provenance of the *S. hispanica* seeds.

Although, due to its nutritional interest, much attention is paid to the tocopherol fraction, according to Suzuki et al. (1993) [29], tocotrienols exhibit antioxidant and antiradical activities superior to those of their corresponding tocopherol homologues. In relation to this, the species belonging to the *Thymus* genus contemplated in this study, i.e., *T*. *vulgaris*, *T*. *zygis* and *T*. *hyemalis* exhibited the highest levels of γ-tocotrienol of 18.8, 11.3 and 9.8 mg/100 g of oil, respectively. *T. hyemalis* (winter thyme) merits special attention since in these seeds, three tocotrienol homologues, i.e., the δ, γ and α forms, were quantified. This should encourage the use of these seeds, considering that, according to Serbinova and Packer (1994) [30], α-tocotrienol has 40–60 times greater antioxidant power than α-tocopherol in liver microsomes.

### 3.3. Phenolic Profile

The screening of the seed oils phenolic profile was carried out by means of HPLC coupled to a diode array detector. The chromatographic analysis allowed the identification of three major phenolic monoterpenes carvacrol, anethol and thymol (Table 3) along with 17 minor polyphenolic components, described in Table 4.

Spanish oregano (*T. capitata*) had, by far, the highest concentration of carvacrol (3355.3 mg/100 g of seed oil), followed by oregano (284.3 mg/100 g of seed oil); statistically, these values differed significantly from each other and from those of the other seed oils analysed. Thymol was quantified as the major phenolic terpene in red thyme seed oil (417.8 mg/100 g of seed oil), which was expected since this was the major component identified previously in the essential oil extracted from its flowers and leaves [31]. This situation can be extrapolated to Spanish oregano and oregano, in light of the fact that carvacrol represents more than 70% of their essential oil volatile profiles [32,33].

Contrastingly, the presence of polyphenols in the seed oil extracts was scarce, and in rosemary, Spanish sage, spike lavender, lavender, red thyme, winter thyme and common thyme only, a few polyphenolic components were detected and quantified. In line with these findings, Fernández-Lopez et al. (2018) [34] reported the quantification of polyphenols at low concentrations in chia seed oil (0.02 mg GAE/g), implying that this fraction remained in the by-product of the seed after the extraction of oil. This would justify why, in spite of chia being an important source of polyphenols, none of these components were detected in its oil.

The occurrence of polyphenols in the seed oil of these species, to the best of our knowledge, is reported for the first time here (Table 4). Thus, it is interesting to point out the detection of 12-methyl carnosic acid (14.7 mg/100 g of seed oil) as the major diterpene quantified in rosemary seed oil, since it is well known that carnosic acid and carnosol are the two major diterpenic components in rosemary leaf extracts [22].

Common thyme and red thyme were also characterized by the presence of diterpenes in their oil polyphenolic fractions, 7-methyl rosmanol, 19.2 and 11.7 mg/100 g seed oil, respectively, being the major polyphenol identified and quantified along with naringenin and eriodictyol.

Spanish sage was the species under study with the richest polyphenolic profile in its seed oil. Salvigenin (10.5 mg/100 g seed oil) was the most-abundant polyphenolic component identified and quantified in this oil, followed by carnosol, apigenin, cirsimaritin, and eupatorin. Salvigenin is a member of the class of compounds known as 7-o-methylated flavonoids and has also been described as the major polyphenolic compound quantified in Spanish sage leaves [35]. 

For spike lavender, the HPLC-DAD chromatographic profile revealed the presence of unknown components that were analysed by means of HPLC–DAD–ESI (Ion Trap)/MSn and UPLC-ESI-QTOF/MS. This analysis yielded a UV-chromatogram (340 nm) with six peaks, all of them showing UV spectra typical of flavonoids (shown in Supplementary Materials). For peaks 1–4, t_R_. 46.9, 47.9, 51.9 and 52.3 min, respectively, their UV spectra exhibited a band II at 268 nm and a band I at 340, 342, 330 and 344 nm, respectively. In the QTOF-MS analysis, these four peaks showed the same deprotonated molecular ion at *m*/*z* 399.0868 ([M − H]^−^, C_24_H_15_O_6_) and in their MS2 fragmentations all of them yielded an ion at *m*/*z* 269.0455, which coincides with that of the deprotonated ion of apigenin ([apigenin − H]^−^, C_15_H_9_O_5_). This fragmentation suggests that these compounds can be described as apigenin derivatives isomers. In addition to this, the loss of the fragment C_9_H_6_O_1_ (cinnamic acid, C_9_H_8_O_2_) denotes that compounds 1–4 could be labelled tentatively as cinnamoyl-apigenin derivatives isomers. Another possibility that needs to be considered is that they can also be named as derivatives of calomelanol D (C_24_H_16_O_7_, substituted apigenin) with one less atom of oxygen that is deoxy-calomelanol D isomers. The other two peaks detected (5 and 6, at Rt 53.6 and 55.0 min, respectively) showed similar UV spectra (267 and 348 and 267 and 342 nm, respectively) when compared to compounds 1–4. The exact mass of the deprotonated molecular ions from both components at *m*/*z* 413.0656 ([M − H]^−^, C_24_H_13_O_7_) leads to the consideration of these components as calomelanol D derivatives with two atoms of hydrogen less—that is, with an additional double bond. Moreover, in the MS2 fragmentation, from both components, the loss of a CO group was observed, giving rise to the ion at *m*/*z* 385.0708 (C_23_H_13_O_6_). From this and considering that they could be apigenin derivatives, due to the UV band II spectra (267 nm) and the presence of apigenin derivatives in these extracts (compounds 1–4), they could be substituted by a C_9_H_4_O_2_ radical, possibly a hydroxycoumarin C_9_H_6_O_3_. This suggests that these components could also be described as apigenin-hydroxycoumarin isomers. These results confirm, once again, the high potential of these seed oils as food ingredients, since in all of them phenolic monoterpenes and/or polyphenolic components have been quantified.

### 3.4. Antioxidant Activity

The results concerning the antioxidant power of these seed oils are shown in Table 5. Among the antioxidant capacities of the seed oils analysed, determined in three in vitro spectrophotometric assays, the highest corresponded to Spanish oregano, followed by oregano, both of which showed statistically significant differences (*p* < 0.05) with respect to the other oils. In this case, the presence of carvacrol, a phenolic monoterpene with high antioxidant power [36] along with a high total tocopherol content in the oils, could justify this, since no polyphenolic compounds were detected in these seed oil extracts. Among the oils in which a polyphenolic fraction was determined, the one extracted from spike lavender seeds stands out since in the ORAC test, it exhibited a high antioxidant power, significantly higher (*p* < 0.05) than those of the other oils under study. Differences found among the three in vitro antioxidant tests, ORAC, ABTS^•+^ and DPPH^•^, are directly related to the chemical principles upon which they are based. This underlines the importance of reporting more than one in vitro antioxidant capacity assay, especially when the main objective is to know the possible health-promoting activity in vivo of these seed oils [37].

## 4. Conclusions

The present study provides knowledge about the potential use of some commercial Lamiaceae seed species as food ingredients. In this regard, seeds from summer savory and winter thyme could be postulated as new alternatives in the production of vegetable lipids. In addition, seeds from winter and summer savory, lavender, spike lavender and oregano are important sources of ω-3 PUFA, as are seed oils from sage and rosemary for ω-6 PUFA. The total tocopherol contents of all the oil seeds under study, with the exception of rosemary, exceeded that detected in commercial chia seeds. The oils with the highest antioxidant capacities corresponded to Spanish oregano and oregano, in which carvacrol was the major phenolic monoterpene quantified. The presence of cinnamoyl-apigenin derivatives and hydroxycoumarin-apigenin derivatives in spike lavender is described for the first time. These derivatives confer a high antioxidant capacity on this seed oil species, according to the ORAC assay. These results confirm the potential of these unexploited seeds as a new source of bioactive components with nutritional interest.

## Figures and Tables

**Table 1:** Absolute quantitative fatty acid profile in the Lamiaceae seeds under study (g/100 g of oil).

	Chia	Summer Savory	Winter Savory	Basil	Oregano	Rosemary	Spanish Sage	Spike Lavender	Lavender	Spanish Oregano	Winter Thyme	Red Thyme	Common Thyme
C16:0	5.3 ± 0.10 ^de^	2.6 ± 0.42 ^a^	2.9 ± 0.05 ^ab^	4.5 ± 0.18 ^d^	3.0 ± 0.05 ^ab^	5.2 ± 0.54 ^de^	5.1 ± 0.40 ^e^	3.3 ± 0.04 ^bc^	3.5 ± 0.13 ^bc^	3.8 ± 0.12 ^d^	2.6 ± 0.07 ^a^	2.9 ± 0.10 ^ab^	2.5 ± 0.02 ^a^
C18:0	2.5 ± 0.04 ^fe^	1.0 ± 0.13 ^a^	0.9 ± 0.01 ^a^	2.2 ± 0.13 ^f^	1.3 ± 0.01 ^cd^	2.6 ± 0.26 ^e^	1.5 ± 0.13 ^de^	1.2 ± 0.02 ^abc^	1.1 ± 0.02 ^ab^	1.6 ± 0.05 ^e^	1.1 ± 0.02 ^abc^	1.2 ± 0.04 ^bcd^	1.1 ± 0.02 ^abc^
C18:1 ω-9	5.9 ± 0.05 ^bc^	4.1 ± 0.65 ^ab^	7.6 ± 0.06 ^cde^	7.0 ± 0.26 ^cd^	3.9 ± 0.10 ^a^	16.2 ± 1.79 ^f^	16.3 ± 1.03 ^f^	9.0 ± 0.62 ^e^	8.9 ± 0.19 ^de^	8.8 ± 0.42 ^de^	5.8 ± 0.10 ^bc^	7.2 ± 0.015 ^cde^	4.9 ± 0.04 ^ab^
C18:1 ω-7 *	0.7 ± 0.02 ^cd^	0.7 ± 0.09 ^de^	0.8 ± 0.02 ^de^	0.6 ± 0.04 ^bc^	0.6 ± 0.01 ^bc^	0.5 ± 0.06 ^bc^	1.0 ± 0.10 ^f^	0.3 ± 0.00 ^a^	0.5 ± 0.02 ^b^	0.7 ± 0.16 ^e^	0.5 ± 0.01 ^b^	0.6 ± 0.01 ^bc^	0.5 ± 0.02 ^b^
C17:3 ω-3 *	n.d.	n.d.	n.d.	n.d.	n.d.	n.d.	n.d.	n.d.	n.d.	n.d.	1.0 ± 0.02 ^c^	0.6 ± 0.02 ^b^	1.8 ± 0.02 ^d^
C18: 2 ω-6	17.3 ± 0.35 ^bcd^	9.6 ± 0.43 ^d^	12.5 ± 0.09 ^abc^	17.1 ± 0.82 ^bcd^	20.2 ± 0.40 ^d^	50.0 ± 5.31 ^e^	52.2 ± 5.52 ^e^	9.6 ± 0.07 ^a^	11.0 ± 0.16 ^ab^	18.1 ± 0.25 ^cd^	10.4 ± 0.18 ^a^	11.5 ± 0.039 ^ab^	10.4 ± 0.06 ^a^
C18:3 ω-3 *^1^	2.0 ± 0.04 ^c^	2.2 ± 0.40 ^c^	2.2 ± 0.07 ^c^	2.2 ± 0.18 ^dc^	2.3 ± 0.16 ^c^	n.d	n.d	2.0 ± 0.02 ^c^	2.3 ± 0.03 ^c^	2.0 ± 0.17 ^c^	2.1 ± 0.31 ^c^	2.0 ± 0.08 ^c^	1.5 ± 0.03 ^b^
C18:3 ω-3^2^	42.5 ± 1.15 ^e^	48.3 ± 5.80 ^fg^	50.2 ± 0.53 ^g^	37.4 ± 1.96 ^d^	43.2 ± 1.00 ^e^	1.8 ± 0.14 ^a^	1.0 ± 0.06 ^a^	46.5 ± 0.51 ^f^	49.8 ± 0.93 ^g^	34.3 ± 1.16 ^c^	37.5 ± 0.46 ^d^	35.3 ± 1.15 ^cd^	29.1 ± 0.17 ^b^
C20:0	0.2 ± 0.00 ^e^	0.1 ± 0.00 ^a^	0.1 ± 0.0 ^a^	0.1 ± 0.0 ^b^	0.1 ± 0.00 ^b^	0.1 ± 0.01 ^c^	0.1 ± 0.00 ^a^	0.1 ± 0.0 ^b^	0.1 ± 0.00 ^b^	0.2 ± 0.00 ^d^	0.1 ± 0.00 ^b^	0.1 ± 0.00 ^b^	0.1 ± 0.00 ^b^
C18:3 ω-3*^3^	n.d.	n.d.	n.d.	n.d	n.d.	n.d.	n.d.	n.d.	n.d.	n.d	3.9 ± 0.03 ^c^	1.9 ± 0.12 ^b^	4.7 ± 0.17 ^d^
SFA	8.0 ± 0.15 ^f^	3.7 ± 0.5 ^ab^	3.9 ± 0.07 ^abc^	6.8 ± 0.31 ^e^	4.4 ± 0.06 ^abc^	7.9 ± 0.82 ^f^	6.7 ± 0.53 ^e^	4.6 ± 0.06 ^c^	4.7 ± 0.15 ^bc^	5.6 ± 0.17 ^d^	3.8 ± 0.07 ^abc^	4.2 ± 0.14 ^abc^	3.7 ± 0.02 ^a^
MUFA	6.6 ± 0.04 ^bcd^	4.8 ± 0.74 ^ab^	8.4 ± 0.06 ^def^	7.6 ± 0.29 ^cde^	4.5 ± 0.11 ^a^	16.7 ± 1.84 ^g^	17.3 ± 1.14 ^g^	9.3 ± 0.62 ^ef^	9.4 ± 0.20 ^ef^	9.5 ± 0.35 ^f^	6.3 ± 0.11 ^abc^	7.8 ± 0.16 ^cdef^	5.4 ± 0.06 ^ab^
PUFA	61.8 ± 1.54 ^cdef^	60.1 ± 9.37 ^g^	64.9 ± 0.63 ^efg^	56.7 ± 2.94 ^bcd^	65.7 ± 1.54 ^fg^	51.8 ± 5.34 ^ab^	53.2 ± 5.53 ^ab^	58.1 ± 0.55 ^bcde^	63.1 ± 1.07 ^defg^	54.4 ± 2.13 ^abc^	54.9 ± 0.30 ^abc^	51.3 ± 1.76 ^ab^	47.5 ± 0.16 ^a^
PUFA ω-3	44.5 ± 1.19 ^d^	50.5 ± 6.16 ^ef^	52.4 ± 0.59 ^f^	39.6 ± 2.12 ^c^	45.5 ± 1.15 ^d^	1.8 ± 0.14 ^a^	1.0 ± 0.06 ^a^	48.5 ± 0.49 ^e^	52.1 ± 0.93 ^f^	36.3 ± 1.10 ^b^	44.5 ± 0.22 ^d^	39.8 ± 1.37 ^c^	37.1 ± 0.21 ^bc^
PUFA ω-6	17.3 ± 0.35 ^bcd^	9.6 ± 3.20 ^d^	12.5 ± 0.09 ^abc^	17.1 ± 0.82 ^bcd^	20.2 ± 0.40 ^d^	50.0 ± 5.31 ^e^	52.2 ± 5.52 ^e^	9.6 ± 0.07 ^a^	11.0 ± 0.17 ^ab^	18.1 ± 1.03 ^cd^	10.4 ± 0.18 ^a^	11.5 ± 0.39 ^ab^	10.4 ± 0.06 ^a^
PUFA ω-9	5.9 ± 0.05 ^bc^	4.1 ± 3.20 ^ab^	7.6 ± 0.06 ^cde^	7.0 ± 0.26 ^cd^	3.9 ± 0.10 ^a^	16.2 ± 1.79 ^f^	16.3 ± 1.03 ^f^	9.0 ± 0.62 ^e^	8.9 ± 0.19 ^de^	8.8 ± 0.42 ^de^	5.8 ± 0.10 ^bc^	7.2 ± 0.15 ^cde^	4.9 ± 0.04 ^ab^

Results are expressed as means (*n* = 9). The different lower-case letters (a–f) in the same row indicate significantly different values (*p* < 0.05). SFA: sum of all saturated fatty acids; MUFA: sum of all monounsaturated fatty acids; PUFA: sum of all polyunsaturated fatty acids; ω-3: sum of all ω-3 polyunsaturated fatty acids; ω-6: sum of all ω-6 polyunsaturated fatty acids; ω-9: sum of all ω-9 polyunsaturated fatty acids. * Tentative identification. n.d. not detected. C16:0 palmitic acid; C18:0 stearic acid; C18:1 ω-9 Z (9) oleic acid; C18:1 ω-7 Z (11) vaccenic acid *; C17:3 ω-3 (8,11,14) heptadecatrienoic acid *; C18: 2 ω-6 Z (9,12) linoleic acid; C18:3 ω-3*^1^E (9, 12, 15) octadecatrienoic acid; C18:3 ω-3^2^ cis (9, 12, 15) α-linolenic acid; C20:0 arachidic acid; C18:3 ω-3*^3^ (9, 12, 15) 2-Hydroxy octadecatrienoic acid.

**Table 2:** Tocopherol content in the Lamiaceae seed oils (mg/100 g of oil).

	Chia	Summer Savory	Winter Savory	Basil	Oregano	Rosemary	Spanish Sage	Spike Lavender	Lavender	Spanish Oregano	Winter Thyme	Red Thyme	Common Thyme
Quinone tocopherol	n.d.	n.d.	0.4 ± 0.02 ^b^	n.d.	1.1 ± 0.04 ^d^	n.d.	7.1 ± 0.04 ^f^	2.3 ± 0.02 ^e^	1.0 ± 0.02 ^c^	n.d.	n.d.	n.d.	n.d.
δ tocopherol	2.0 ± 0.02 ^c^	2.4 ± 0.02 ^f^	1.3 ± 0.02 ^a^	3.2 ± 0.03 ^g^	2.3 ± 0.03 ^e^	1.5 ± 0.02 ^b^	3.4 ± 0.02 ^h^	2.2 ± 0.02 ^c^	1.5 ± 0.01 ^b^	3.6 ± 0.04 ^i^	2.4 ± 0.02 ^f^	4.0 ± 0.02 ^j^	3.2 ± 0.02 ^g^
γ tocopherol	56.4 ± 0.88 ^f^	67.9 ± 0.06 ^j^	56.8 ± 0.06 ^f^	110.3 ± 0.19 ^k^	68.1 ± 0.20 ^i^	38.5 ± 0.06 ^a^	49.4 ± 0.02 ^c^	81.5 ± 0.11 ^j^	61.4 ± 0.14 ^g^	64.7 ± 0.03 ^h^	52.3 ± 0.03 ^d^	54.0 ± 0.07 ^e^	46.0 ± 0.16 ^b^
α tocopherol	n.d.	n.d.	2.3 ± 0.04 ^c^	n.d.	4.6 ± 0.06 ^e^	2.3 ± 0.03 ^c^	n.d.	n.d.	1.9 ± 0.04 ^b^	n.d.	n.d.	n.d.	2.5 ± 0.05 ^d^
δ tocotrienol	n.d.	n.d.	n.d.	n.d	n.d.	n.d.	n.d.	0.1 ± 0.02 ^b^	n.d.	n.d	0.4 ± 0.02 ^c^	n.d.	n.d.
γ tocotrienol	n.d.	03 ± 0.02 ^b^	n.d.	n.d.	0.9 ± 0.09 ^d^	0.5 ± 0.04 ^c^	5.9 ± 0.04 ^f^	0.7 ± 0.05 ^c^	0.9 ± 0.05 ^d^	1.7 ± 0.05 ^e^	9.8 ± 0.08 ^g^	11.3 ± 0.14 ^h^	18.8 ± 0.10 ^i^
α tocotrienol	n.d.	n.d.	n.d.	0.3 ± 0.03 ^b^	n.d.	n.d.	n.d.	n.d.	n.d.	n.d.	0.6 ± 0.07 ^c^	n.d.	n.d.
Total tocopherols	58.4 ± 0.90 ^b^	70.6 ± 0.05 ^g^	60.8 ± 0.02 ^c^	113.8 ± 0.023 ^j^	77.0 ± 0.36 ^h^	42.8 ± 0.04 ^a^	65.8 ± 0.02 ^d^	86.8 ± 0.09 ^i^	66.7 ± 0.15 ^e^	70.0 ± 0.09 ^g^	65.5 ± 0.10 ^d^	69.3 ± 0.11 ^f^	70.5 ± 0.20 ^g^

Results are expressed as means (*n* = 9). The different lower-case letters (a–h) in the same row indicate significantly-different values (*p* < 0.05). n.d. not detected.

**Table 3:** Phenolic monoterpenes quantified in the seed oils (mg/100 g of oil).

	Anethol	Carvacrol	Thymol
Chia	n.d.	n.d.	n.d.
Summer savory	n.d.	6.9 ± 0.46 ^a^	4.9 ± 0.30 ^a^
Winter savory	n.d.	7.2 ± 0.53 ^a^	0.9 ± 0.06 ^a^
Basil	n.d.	5.13 ± 0.42 ^a^	11.8 ± 0.82 ^a^
Oregano	48.0 ± 3.36 ^d^	284.3 ± 8.33 ^b^	214.8 ± 5.36 ^d^
Rosemary	12.2 ± 1.26 ^b^	6.2 ± 0.68 ^a^	6.0 ± 0.3 ^a^
Spanish sage	n.d.	2.0 ± 0.2 ^a^	7.6 ± 5.78 ^a^
Spike lavender	n.d.	n.d.	n.d.
Lavender	n.d.	n.d.	n.d.
Spanish oregano	20.7 ± 3.05 ^c^	3355.3 ± 91.26 ^c^	23.5 ± 1.8 ^b^
Winter thyme	n.d.	22.7 ± 2.08 ^a^	2.6 ± 0.25 ^a^
Red thyme	22.3 ± 2.52 ^c^	50.6 ± 1.40 ^a^	417.8 ± 10.44 ^e^
Common thyme	n.d.	23.6 ± 1.27 ^a^	171.3 ± 7.53 ^c^

Results are expressed as means (*n* = 9). The different lower-case letters (a–e) in the same column indicate significantly different values (*p* < 0.05). n.d. not detected.

**Table 4:** Major polyphenolic compounds quantified in the Lamiaceae seed oils (mg/100 g of oil).

	Rosemary	Spanish	Spike	Lavender	Winter	Red	Common Thyme
		Sage	lavender		Thyme	Thyme	
Eriodictyol	n.d.	n.d.	n.d.	n.d.	0.2 ± 0.02 ^b^	1.5 ± 0.2 ^c^	1.6 ± 0.17 ^c^
Luteolin	n.d.	n.d.	n.d.	n.d.	n.d.	n.d.	n.d.
Naringenin	n.d.	n.d.	n.d.	0.41 ± 0.02 ^b^	0.4 ± 0.03 ^b^	4.2 ± 0.3 ^c^	2.7 ± 0.14 ^c^
Apigenin	0.6 ± 0.04 ^a^	4.0 ± 0.25 ^b^	n.d.	n.d.	n.d.	n.d.	n.d.
Cinnamoyl-apigenin derivate 1	n.d.	n.d.	162.3 ± 4.04 ^b^	n.d.	n.d.	n.d.	n.d.
Cinnamoyl-apigenin derivate 2	n.d.	n.d.	1.0 ± 0.10 ^b^	n.d.	n.d.	n.d.	n.d.
Cinnamoyl-apigenin derivate 3	n.d.	n.d.	4.1 ± 0.20 ^b^	n.d.	n.d.	n.d.	n.d.
Cinnamoyl-apigenin derivate 4	n.d.	n.d.	0.5 ± 0.01 ^b^	n.d.	n.d.	n.d.	n.d
Cirsimaritin	1.5 ± 0.01 ^b^	2.9 ± 0.03 ^c^	n.d.	1.1 ± 0.08 ^b^	n.d.	n.d.	n.d.
Eupatorin	n.d.	1.9 ± 0.21 ^b^	n.d.	n.d.	n.d.	n.d.	n.d.
Genkwanin	2.6 ± 0.08 ^b^	n.d.	n.d.	n.d.	n.d.	n.d.	n.d.
Salvigenin	n.d.	10.5 ± 1.27 ^b^	n.d.	n.d.	n.d.	n.d.	n.d.
7-methyl rosmanol	n.d.	n.d.	n.d.	6.4 ± 0.28 ^b^	n.d.	11.7 ± 0.73 ^c^	19.2 ± 0.31 ^d^
Carnosol	n.d.	5.0 ± 0.15 ^b^	n.d.	n.d.	n.d.	n.d.	n.d.
12-methyl carnosic acid	14.7 ± 0.33 ^b^	n.d.	n.d.	n.d.	n.d.	n.d.	n.d.
Hydroxycoumarin-apigenin derivate 5	n.d.	n.d.	3.2 ± 0.48 ^b^	n.d.	n.d.	n.d.	n.d.
Hydroxycoumarin-apigenin derivate 6	n.d.	n.d.	2.9 ± 0.04 ^b^	n.d.	n.d.	n.d.	n.d.

Results are expressed as means (*n* = 9). The different lower-case letters (a–d) in the same column indicate significantly different values (*p* < 0.05). n.d. not detected.

**Table 5:** Seed oil antioxidant activities (µmol Trolox/g oil).

	ABTS^•+^	DPPH^•^	ORAC
Chia	1.21 ± 0.10 ^a^	1.3 ± 0.00 ^a^	13.2 ± 0.62 ^a^
Summer savory	2.5 ± 0.16 ^ab^	1.8 ± 0.04 ^ab^	88.7 ± 1.84 ^abcd^
Winter savory	2.2 ± 0.03 ^ab^	1.8 ± 0.02 ^ab^	26.3 ± 0.56 ^a^
Basil	3.3 ± 0.17 ^bc^	2.8 ± 1.16 ^abc^	15.5 ± 1.37 ^a^
Oregano	34.1 ± 1.41 ^f^	4.8 ± 0.10 ^f^	121.4 ± 4.72 ^bcd^
Rosemary	4.5 ± 0.11 ^cd^	3.8 ± 0.79 ^de^	33.4 ± 1.59 ^ab^
Spanish sage	6.8 ± 0.20 ^e^	3.0 ± 0.02 ^cd^	23.1 ± 1.22 ^a^
Spike lavender	5.1 ± 0.11 ^d^	2.4 ± 0.02 ^bc^	769.8 ± 34.37 ^e^
lavender	1.5 ± 0.16 ^a^	1.6 ± 0.03 ^ab^	65.4 ± 5.73 ^abc^
Spanish oregano	62.6 ± 1.70 ^g^	9.6 ± 0.04 ^g^	1219.9 ± 117.25 ^f^
Winter thyme	3.1 ± 0.16 ^bc^	1.7 ± 0.02 ^ab^	70.2 ± 6.82 ^abcd^
Red thyme	4.6 ± 0.16 ^cd^	3.9 ± 0.10 ^de^	153.3 ± 2.14 ^cd^
Common thyme	2.7 ± 0.22 ^ab^	4.1 ± 0.05 ^e^	161.0 ± 8.36 ^d^

Results are expressed as means (*n* = 9). The different lower-case letters (a–g) in the same column indicate significantly different values (*p* < 0.05).

## Supplementary Materials

The following are available online at https://www.mdpi.com/2076-3921/9/6/510/s1, Figure S1: HPLC-UV (340 nm) profile of polyphenolic compounds identified in *L*. *latifolia* oil seeds and Figure S2: (A) total ion chromatogram (TIC) of phenolic compounds from *L*. *latifolia* oil seeds. Extracted ion chromatogram (EIC) of [M − H]^−^ (*m*/*z*): (B) 399.0868 and (C) 413.0656.
