# Supplementary material for: Seed Oil from Mediterranean Aromatic and Medicinal Plants of the Lamiaceae Family as a Source of Bioactive Components with Nutritional
Source: Antioxidants (Basel). 2020 Jun 10;9(6):510. doi: 10.3390/antiox9060510 (PMC7346175; doi:10.3390/antiox9060510)
Supplement: Supplementary file 1 [file antioxidants-09-00510-s001.pdf]

Seed Oil from Mediterranean Aromatic and Medicinal Plants of the Lamiaceae as a Source of Bioactive Components with Nutritional

María Quílez[1], Federico Ferreres[2], Santiago López-Miranda[2], Eva Salazar[2], María J. Jordán[1*]

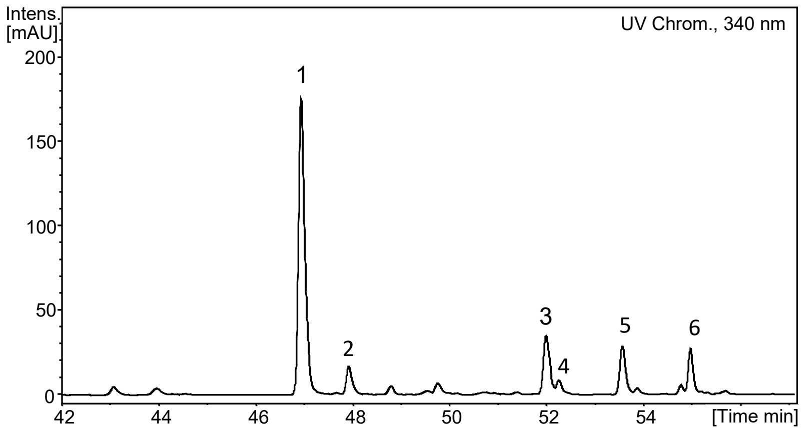

Figure S1. HPLC-UV (340 nm) profile of polyphenolic compounds identified in *L. latifolia* oil seeds.

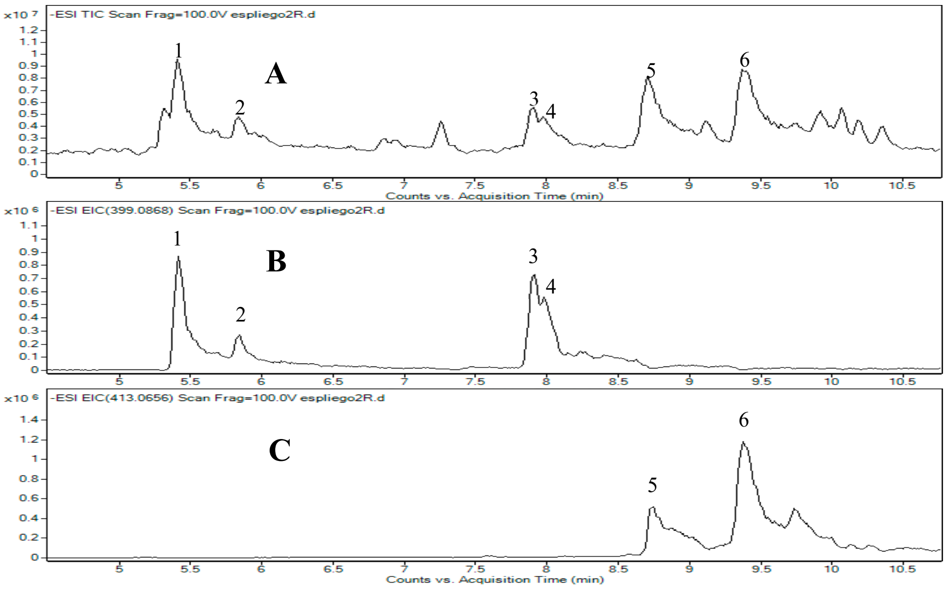

Figure S2. A) Total Ion Chromatogram (TIC) of phenolic compounds from L. latifolia oil seeds. Extracted Ion Chromatogram (EIC) of [M-H] - (m/z): B) 399.0868. C) 413.0656.
